# Evidence of Mixed Selection Acting on the MHC Class II *DQA* Gene in Captive Thai Elephant Populations

**DOI:** 10.3390/genes16101180

**Published:** 2025-10-10

**Authors:** Trifan Budi, Marie Roselle Enguito, Worapong Singchat, Thitipong Panthum, Ton Huu Duc Nguyen, Aingorn Chaiyes, Narongrit Muangmai, Darren K. Griffin, Prateep Duengkae, Kornsorn Srikulnath

**Affiliations:** 1 Animal Genomics and Bioresource Research Unit (AGB Research Unit), Faculty of Science, Kasetsart University, 50 Ngamwongwan, Chatuchak, Bangkok 10900, Thailand; trifan.bu@ku.th (T.B.); worapong.si@ku.th (W.S.); thitipong.pa@ku.th (T.P.); nguyenhuuduc.t@ku.th (T.H.D.N.); aingorn.ch@ku.ac.th (A.C.); ffishnrm@ku.ac.th (N.M.); d.k.griffin@kent.ac.uk (D.K.G.); prateep.du@ku.ac.th (P.D.); 2 School of Biosciences, University of Kent, Canterbury, Kent CT2 7NJ, UK; enguitomarieroselle@gmail.com; 3 Special Research Unit for Wildlife Genomics (SRUWG), Department of Forest Biology, Faculty of Forestry, Kasetsart University, 50 Ngamwongwan, Chatuchak, Bangkok 10900, Thailand; 4 The International Undergraduate Program in Bioscience and Technology, Faculty of Science, Kasetsart University, 50 Ngamwongwan, Chatuchak, Bangkok 10900, Thailand; 5 Department of Fishery Biology, Faculty of Fisheries, Kasetsart University, Chatuchak, Bangkok 10900, Thailand; 6 Biodiversity Center, Kasetsart University (BDCKU), Chatuchak, Bangkok 10900, Thailand

**Keywords:** captive elephants, *DQA* gene, immune health, MHC, selection signature

## Abstract

**Background:** The health and viability of captive elephants, which are central to off-site conservation efforts and health management in Thailand, is threatened by emerging infectious diseases. This is partly due to genetic differences in immune-related genes, especially in the major histocompatibility complex (MHC) and, among these, loci such as *DQA* play a crucial role in immune surveillance. Data pertaining to MHC polymorphisms in elephants are scarce, and thus this study investigated such polymorphisms and selection signatures in a partial fragment of exon 2 of the MHC Class II *DQA* gene. **Methods:** The approach we used targeted next-generation sequencing and diversity analyses of individuals from three captive elephant camps in Northern Thailand. **Results:** Eight alleles containing 11 SNPs were identified in the exon 2 fragment, encompassing both silent and missense mutations, some of which may influence immune function. Notably, the allele Elma-DQA*TH3, which is identical to Loaf-DQA*01 and Elma-DQA*01, previously reported as the most common alleles in *Loxodonta* and *Elephas*, was found at low frequencies. This shift may reflect local selective pressures that shape MHC allele distributions. Evidence of mixed selection (both positive and balancing) was detected in the partial fragment of *DQA* exon 2, suggesting a dynamic interplay between evolutionary forces. Positive selection likely reflects an adaptation to emerging or locally prevalent pathogens, whereas balancing selection maintains allelic diversity over time to enable a broad immunological response. **Conclusions:** Our findings reveal immunogenetic variations in captive Thai elephants, and provides insights into host–pathogen interactions that inform conservation and health strategies with the aim of improving disease resilience.

## 1. Introduction

The major histocompatibility complex (MHC) genes play a fundamental role in vertebrate immunity by encoding cell surface glycoproteins that bind to, and present, foreign antigens for recognition by immune cells [1]. MHC class II genes are highly polymorphic, and each allele enables recognition of different antigens [2,3]. Individuals or populations with a higher diversity of MHC loci are better equipped to cope with a broad spectrum of pathogens [4,5]. Loci such as *DQA*, which encode part of the MHC class II DQ heterodimer, are highly polymorphic, and play a crucial role in immune surveillance by presenting peptides to T cells [6]. This functional role highlights the importance of maintaining genetic variability at these loci [6]. Extensive diversity at MHC loci has been widely attributed to balancing selection [3,7,8] and MHC allelic lineages are often retained across species (trans-species polymorphism) [9,10]. The codons encoding the antigen-binding regions, particularly in the second exon of class II genes, exhibit elevated rates of nonsynonymous relative to synonymous substitutions [11]. Diversity patterns vary, however, between exons within the same locus and across species, reflecting locus- or taxon-specific selection pressures. MHC, recognized for its role in pathogen detection, is used as a biomarker for assessing functional genetic diversity in wild or captive populations, including elephants [12,13]. Therefore, understanding MHC variations is essential to predict population-level responses to emerging infectious diseases.

The Asian elephant (*Elephas maximus*) is an ecologically important umbrella and flagship species that has been classified as ‘Endangered’ by the IUCN [14]. It is found in Thailand, where it has remarkable cultural significance. Currently, 6500–7500 individuals remain, of which 3500–3800 are captive and 3000–3700 occur in 69 protected areas [15]. Major threats include habitat loss and fragmentation, which can lead to intensified human–elephant conflict [14,16,17,18]. In addition to these pressures, disease poses a significant threat to elephant populations; contributes to morbidity and mortality that can, in turn, compromise immune function and overall population fitness [19,20]. Most captive elephants in Thailand are used for tourism, especially in the northern region; they contribute significantly to the national economy and thereby support the maintenance of overall elephant populations [21]. Although free-ranging populations face severe conservation challenges, captive populations are considered relatively stable [15,21], albeit with reproductive difficulties and health problems that remain prevalent in captivity [22,23]. Captive elephants often exhibit low fecundity, behavioral incompatibilities between the sexes and limited breeding success. Moreover, diseases such as elephant endotheliotropic herpesvirus (EEHV) hemorrhagic disease have caused substantial mortality among captive individuals [24,25]. Other infectious diseases include encephalomyocarditis, salmonellosis and anthrax [26,27,28] and tuberculosis caused by *Mycobacterium tuberculosis*, which is transmitted from humans to elephants [29]. All of these are emerging concerns for Asian elephants and may further compromise the long-term sustainability of captive populations. Differences in disease susceptibility and severity among elephant populations are influenced by immune-related genes, especially MHC genes. This is true for shared pathogens, including parasites, foot-and-mouth disease virus, rabies, tuberculosis, trypanosomiasis and septicemia [30,31]. The risk of interspecies disease transmission is increased by the expansion of agricultural land use, which brings elephants into close contact with livestock [32,33]. Such cross-species transmission events often result in severe disease outcomes in novel hosts [34,35]. These ongoing health and reproductive issues may have significant economic implications for the tourism industry as it depends on captive elephants [15,21,23].

Data on MHC gene diversity in captive elephants in Thailand, which are required to predict responses to emerging disease threats, are currently lacking. The assessment of MHC variability is essential, especially considering climate change, which can influence pathogen survival, transmission, and host susceptibility [33,35]. Previous studies on Thai captive elephants have revealed substantial genetic variation in neutral microsatellite loci and mtDNA, indicating considerable genetic diversity among populations [36,37,38,39]. This suggests the existence of similar diversity at the MHC *DQA* gene, potentially maintained by long-term balancing selection mechanisms such as trans-species polymorphism [3,7,8,9,10,11]. The aim of present study was to evaluate the polymorphisms and signatures of selection in a partial fragment of exon 2 of the *DQA* gene. To do this, we used targeted next-generation sequencing and genetic diversity analyses across elephants from three captive camps in Northern Thailand.

## 2. Materials and Methods

### 2.1. Sample Collection and DNA Extraction

A total of 123 individual elephants were sampled in the National Elephant Institute of Thailand (NEI, Lampang; 18°21′35.5″ N 99°14′52.9″ E, *n* = 39), Maetaeng Elephant Park (MEP, Chiang Mai; 19°11′54.2″ N 98°53′14.9″ E, *n* = 40), and Baan Chang Elephant Park (BCEP, Chiang Mai; 19°07′29.0″ N 98°53′38.7″ E, *n* = 41) between October 2020 and November 2021. The farm owners determined the number of specimens collected per camp. Blood samples were obtained from the jugular vein using 18-gauge needles attached to 5 mL disposable syringes containing 10 mM ethylenediaminetetraacetic acid (EDTA). All the elephants were immediately released into the same area after sample collection. Sample collection permits were granted by the Kasetsart University, NEI (approval no. 1400/476), MEP (no. 6501.0901/3349), and BCEP (no. 6501.0901/3968). Detailed information on the sampled individuals is provided in Table S1. Total genomic DNA was extracted from blood following the standard phenol-chloroform-isoamyl alcohol protocol described by Srikulnath et al. [40]. DNA quality and quantity were assessed by 1% agarose gel electrophoresis and a NanoDrop 2000 spectrophotometer (Thermo Fisher Scientific, Wilmington, DE, USA). All animal care and experimental procedures were approved by the Animal Experiment Committee of Kasetsart University (approval no. ACKU63-SCI-017) and were conducted in full compliance with the Regulations on Animal Experiments at Kasetsart University and the ARRIVE guidelines (https://arriveguidelines.org/, accessed on 9 September 2022).

### 2.2. Polymerase Chain Reaction and Illumina^TM^ Short-Read Sequencing

The partial fragment of exon 2 of the *DQA* gene, which is highly variable in mammals [41], was amplified via polymerase chain reaction (PCR) using the primer set MPDQA 1iF (5′-TGGAGATGAGCTGTTCTACGT-3′) and MPDQA 1iR (5′-AGCACAGCTATGTTCCTCAGTC-3′) [42]. The forward primer was modified by adding specific 8 bp individual barcode sequences at the 5′-end (Macrogen Inc., Seoul, Republic of Korea). Each 15 μL of PCR mixture consisted of 50 ng DNA template and 1× Apsalagen buffer containing 1.5 mM MgCl_2_, 0.2 mM dNTPs, 0.5 μM primers, and 0.5 U *Taq* polymerase (Apsalagen Co., Ltd., Bangkok, Thailand). The cycling conditions were as follows: initial denaturation at 94 °C for 5 min, followed by 35 cycles at 94 °C for 30 s, annealing at 58 °C for 30 s, and elongation at 72 °C for 30 s, followed by a final extension at 72 °C for 5 min. PCR products were detected by electrophoresis on a 1% agarose gel. Each sample was run in triplicate to avoid false allele amplification. Ninety-two samples amplified per pool set with each barcode primer were pooled into six pool sets and sent for paired-end short-read sequencing on an Illumina NovaSeq^TM^ 6000 platform (Novogene Co., Ltd., Singapore).

### 2.3. Sequence Quality Control and Processing

Paired-end 250 bp reads were evaluated using FASTQC v0.12.0 [43], retaining only reads with Phred scores (q) >20. Reads were merged, demultiplexed, and filtered to isolate individual amplicons, and *DQA* alleles were assigned to each individual using AmpliSAS [44]. To account for low reads and potential artifacts, the minimum amplicon depth was set to 100. The maximum number of alleles per individual was set to six to account for possible duplications in the *DQA* genes [45]. True alleles were identified using a degree of change (DOC) filter [46] with default parameters for other settings. Allele sequences were validated using BLASTn against the NCBI database (http://blast.ncbi.nlm.nih.gov/Blast.cgi, accessed on 9 September 2023), aligned, translated to amino acids, and checked for the presence of stop codons using Geneious Prime v2024.0.5 (https://www.geneious.com, accessed on 9 September 2024). The allele sequences found in this study were deposited in the National Center for Biotechnology Information (NCBI) (https://www.ncbi.nlm.nih.gov/, accessed on 9 September 2025) (accession numbers: SRX30398467–SRX30398474).

### 2.4. Genetic Diversity and Selection Analysis

Genetic diversity was estimated by calculating the number of alleles (*N*_a_) and nucleotide diversity (*π*) using DnaSP version 6.12 [47]. Neutrality tests based on Tajima’s *D*, Fu and Li’s F*, or Fu and Li’s D*) were performed using DnaSP version 6.12 [47]. Patterns of selection were assessed by estimating the average amount of synonymous (*d*_S_) and nonsynonymous (*d*_N_) substitutions per site (*d*_N_/*d*_S_ (ω) ratio) based on Nei–Gojobori’s method [48] with Jukes–Cantor in Molecular Evolutionary Genetics Analysis (MEGA) 11 [49]. The Pamilo–Bianchi–Li method, which accounts for different rates of transitional and transversional substitutions, was also applied to provide a more realistic estimation of *d_N_*/*d_S_*. A ratio close to 1 indicates neutrality, ω values > 1 indicate positive selection, whereas ω values < 1 indicate purifying selection. Furthermore, site-specific signatures of pervasive selection (acting uniformly across the phylogeny) were inferred using Fast Unconstrained Bayesian AppRoximation (FUBAR) [50] and fixed effect likelihood (FEL) [51] approaches. Episodic positive selection (acting on branch subsets) was detected using a mixed effects model of evolution (MEME) [52]. Codon-specific selection scores were calculated using likelihood ratio tests implemented in the Datamonkey MEME framework, with significance thresholds set at *p* < 0.001. The Y-axis shows −log_10_ *p* values, as directly generated by MEME, which allows visualization of codon-specific differences while ensuring consistency with standard Datamonkey outputs. Episodic diversifying selection at individual sites was identified by MEME, which allows the distribution of *ω* to vary across branches, occasionally resulting in extremely small *p*-values when a strong episodic signal is detected by the likelihood ratio test. All analyses used the default settings on the Datamonkey web server [53], with input trees derived from sequence alignments. Sites with posterior probability > 0.95 (FUBAR) or *p* < 0.05 (FEL, MEME) were considered statistically significant.

### 2.5. Phylogenetic Analysis of the Elephant MHC Class II DQA Gene

To gain insight into the evolutionary history of the *DQA* gene alleles in captive elephants, a phylogenetic tree was constructed based on the Bayesian approach using MrBayes version 3.2.6 [54]. ModelFinder [55] was used to determine the best-fit substitution model based on the lowest Bayesian information criterion (BIC) value. Markov chain Monte Carlo (MCMC) was run to approximate the posterior probabilities of trees, with the setting parameters of four chains, 1,000,000 chain length, 10,000 burn-in period, and sampled every 100 generations. Partial *DQA* allele sequences across elephant species were obtained by retrieving partial fragments of exon 2 using BLASTn (http://blast.ncbi.nlm.nih.gov/Blast.cgi, accessed on 9 September 2023. The phylogenetic tree was visualized using Interactive Tree of Life (iTOL) version 5 [56].

### 2.6. Multiple Sequence Alignment of Elephant MHC Class II DQA and Secondary Structure Prediction

The translated MHC Class II *DQA* alleles obtained in this study were used as queries in BLASTp searches (https://blast.ncbi.nlm.nih.gov/Blast.cgi, accessed on 9 September 2023). BLASTp analyses were conducted using filtering thresholds of >60% sequence similarity and >85% query coverage [57,58]. The resulting sequences, along with those identified in the present study, were aligned using ClustalW in Geneious Prime v2023.0.4 (https://www.geneious.com, accessed on 9 September 2024. The alignment was trimmed to 40 amino acids, and a Bayesian phylogenetic tree was constructed as previously described. Protein secondary structures were predicted using the EMBOSS tool v6.5.7 (http://emboss.sourceforge.net/, accessed on 9 September 2024), applying the Garnier-Osguthorpe-Robson (GOR I) algorithm as implemented in Geneious Prime v2023.0.4 (https://www.geneious.com, accessed on 9 September 2024.

## 3. Results

### 3.1. Polymorphism and Phylogenetics of the Partial Fragment of DQA Exon 2 in Captive Elephants

**Figure 1:**
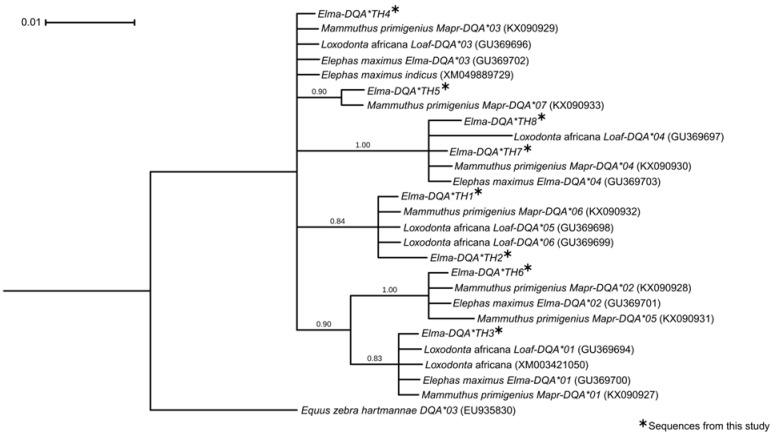
Bayesian phylogenetic tree of partial exon 2 of the *DQA* gene alleles for captive Thai elephants. The values above the branches represent posterior probability. Scale shows substitutions per site.

Nucleotide sequences of 121 bp partial fragments covering exon 2 of *DQA* were obtained by short-read sequencing. Eleven variable sites defining eight alleles were identified. Of these, seven alleles were newly identified, and one allele (Elma-DQA*TH4) was identical to the reference sequences of the Asian elephant (CM065237) and African bush elephant (NC087342). The alleles Elma-DQA*TH1, Elma-DQA*TH5, and Elma-DQA*TH6 were found to be common in this study. Two alleles (Elma-DQA*TH3 and Elma-DQA*TH4) were identified as rare and were restricted to the NEI population. Compared with the reference sequences, the eight alleles exhibited 12 mutational sites in the exon, consisting of five silent mutations and seven missense mutations, including seven transversions and five transitions (Tables S2–S4). The number of alleles per individual ranged from one to five, with a mean *π* value of 0.030 (Table 1). The Bayesian phylogenetic tree revealed a polyphyletic pattern with a lack of distinct clades according to the population and/or species (Figure 1).

### 3.2. Pattern of Selection Within Partial DQA Gene Exon 2 Sequences in Captive Elephants

**Figure 2:**
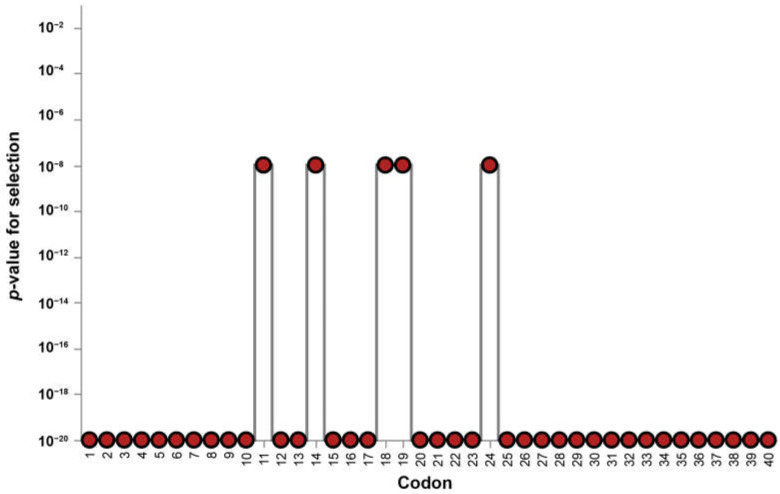
Mixed effects model of evolution (MEME) results showing selection at individual codons in the partial exon 2 of the *DQA* gene. The Y-axis shows the −log_10_(*p*) values generated directly by MEME on the Datamonkey server. Each red dot represents a codon, with significant codons (11, 14, 18, 19, and 24; *p* < 0.001) highlighted as sites under episodic diversifying selection.

Evidence for selection was observed in the partial fragment of the *DQA* exon 2 sequence of captive elephants from three camps in Thailand (Table 2). The mean values for the neutrality tests were positive for Tajima’s *D*, Fu and Li’s D*, and Fu and Li’s *F*. Tajima’s *D* and Fu and Li’s D* values were not significant, whereas Fu and Li’s *F* values were significant. The mean *ω* value estimated by Nei–Gojobori’s method was 3.75, while the Pamilo–Bianchi–Li method yielded a slightly lower overall ratio of 2.8; both indicating positive selection. MEME analysis revealed that the partial *DQA* exon 2 sequences underwent episodic diversifying selection; specifically, five codons (codons 11, 14, 18, 19, and 24) likely underwent diversifying selection (*p* < 0.001) (Figure 2 and Table S5). FEL indicated that the partial *DQA* exon 2 sequences were not under positive or purifying selection (*p* < 0.001), with five codons showing evidence of neutral selection (codons 11, 14, 18, 19, and 24) (Table S6). FUBAR indicated a codon exhibiting a signature of positive selection (codon 18) and one exhibiting negative selection (codon 14) with strong support (*p* = 0.9) (Table S7).

### 3.3. Secondary Structure and Sequence Homology of DQA Alleles

**Figure 3:**
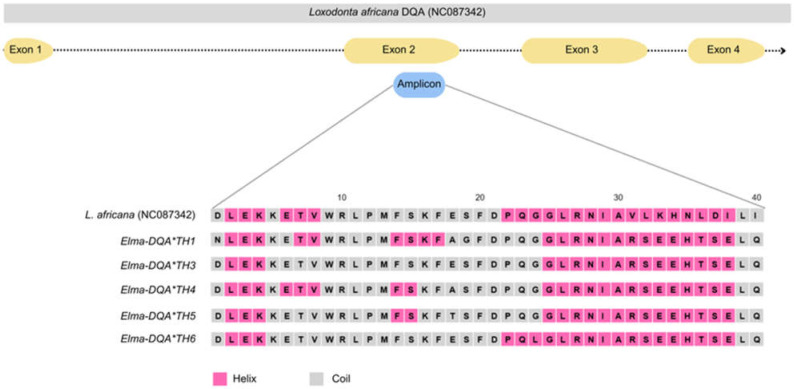
Amino acid sequences of partial exon 2 of the *DQA* gene alleles and prediction of secondary structures of protein in captive Thai elephants.

The 40 amino acid residues of the *DQA* gene from the eight alleles identified in this study exhibited high similarity to *DQA* allele sequences from the family Elephantidae available in the GenBank database, with a mean pairwise similarity exceeding 70%. Protein sequence homology analysis further revealed that the exon 2 region of these alleles shared a high degree of similarity with sequences from Asian elephants, African bush elephants, and wooly mammoths, with query coverage ranging from 75% to 98%. These alleles are located in the α domain of the Class II histocompatibility antigen domain in the antigen-binding region (ABR). Phylogenetic analysis based on the amino acid sequence alleles showed no clustering by species or population in *DQA* alleles of the family Elephantidae (Figure S1). Secondary structure prediction indicated that the region corresponding α domain of the Class II histocompatibility antigen was primarily composed of α-helix and coil structures (Figure 3).

## 4. Discussion

### 4.1. Demographic History and Pathogen Dynamics Drive DQA Polymorphism

The patterns of MHC diversity across species reflect the influence of demography, life history and disease pressure on MHC evolution [41]. As long-lived animals, elephants encounter a wide range of pathogens throughout their lifespan, highlighting the importance of relatively high MHC diversity [41]. In this study, a total of eight alleles, defined by 11 variable sites within exon 2 of the *DQA* gene, were observed. There was greater variation in exon 2 compared to previous reports on African elephants (six alleles), Asian elephants (four alleles), mammoths (5–7 alleles) and other mammalian species such as dolphins (four alleles) [41,42,59,60]. In contrast, the observed variation was lower than that reported for other mammals such as pig-tailed macaques [61] and hares [62,63]. The alleles Elma-DQA*TH1, Elma-DQA*TH5 and Elma-DQA*TH6 were the most frequently observed. Notably, Elma-DQA*TH1 exhibited high sequence similarity to alleles identified in both extant African elephants (Loaf-DQA*05 and Loaf-DQA*06) and extinct mammoths (Mapr-DQA*06). Allele Elma-DQA*TH6 closely resembled the alleles reported in both Asian elephants (Elma-DQA*02) and mammoths (Mapr-DQA*02) [41]. Similarly, Elma-DQA*TH5 was highly similar to allele Mapr-DQA*07 reported previously in mammoths [42]. This suggests that the identified alleles offer a selective advantage through resistance to pathogens and supports the hypothesis of trans-species polymorphism, which involves the maintenance of MHC allelic lineages across species over evolutionary timescales [41]. The allele Elma-DQA*TH3, restricted to NEI and with a low frequency, shares high similarity with the alleles Loaf-DQA*01 and Elma-DQA1*01, which are common in *Loxodonta* and *Elephas*. While Loaf-DQA*01 and Elma-DQA1*01 may confer evolutionary advantages, purifying selection may have occurred in captive Thai populations due to different environmental and pathogen pressures [12,64]. This observation underscores the role of local pathogen-mediated selection in shaping MHC polymorphism [65] and may reflect differences in disease and pathogen dynamics unique to the Thai environment [66,67,68]. Differences in the dominant EEHV types between Thailand and other countries support region-specific pathogen evolution and transmission patterns [24]. Similar geographic structuring of pathogens or immune gene variation has been documented in other species, including Eastern Atlantic gray seals [69], Omei tree frogs [70] and three-spined sticklebacks [71].

The highest DQA variation was observed in the NEI population, which harbored two unique alleles that were absent in MEP and BCEP. This, most likely reflects its distinct demographic history and diverse founder composition, which is, in turn, supported by previous mtDNA and microsatellite data [38,39,40]. MtDNA D-loop analyses placed MEP and BCEP individuals in α and β1 haplogroups, while NEI individuals spanned α, β1, β3, and an unclassified haplogroup, suggesting broader maternal lineage diversity. Similarly, microsatellite data have revealed a mixed gene pool in the NEI population, indicating a more diverse biparental genetic background [38,40]. These findings highlight the influence of demographic history on shaping MHC variability within the NEI population. Alternatively, differences in diversity levels among populations may be attributed to either a difference in the speed of allelic diversification or the time of their persistence in the gene pool [9,62] in response to different local pathogens and/or environmental pressures. These findings, however, should be interpreted with caution as they are based on short sequence fragments from a single locus. More robust insights into MHC diversity in captive Thai elephants require longer sequences, multiple loci and broader sampling across individuals and populations.

### 4.2. Pattern of Selection and Evidence of Trans-Species Polymorphism in the Partial Fragment of DQA Exon 2 in Captive Thai Elephants

The phylogenetic tree based on nucleotide sequences and amino acid residues showed no clear clades by species, supporting the hypothesis of a trans-species allelic polymorphism, as previously proposed for the two elephant genera [41]. These trans-species allelic lineages found in both extant and extinct species may have been maintained over relatively long evolutionary time frames through balancing selection [3,7,8,9,10]. Trans-species allelic polymorphisms in MHC genes are common in mammals, birds, reptiles, amphibians, and fish [10]. The positive neutrality test results suggested a balancing selection [72], further supporting the phylogenetic tree pattern. Two neutrality tests (Tajima’s *D* and Fu and Li’s *D*), however, were insignificant. This potentially indicates that genetic variations in MHC genes in captive Thai elephant populations may result from stochastic processes, such as genetic drift, as has been observed in other mammals [73,74]. Balancing selection is also well documented to occur alongside positive selection in MHC genes [75]. This study also revealed a signature of positive selection: That is, the *d*_N_/*d*_S_ ratio revealed that a partial fragment of the *DQA* exon 2 in captive Thai elephants exhibited positive selection, which was characterized by a higher number of nonsynonymous codons than synonymous changes. The observed high genetic diversity, with multiple alleles present at intermediate frequencies, also supports a positive selection signature within the population [76,77,78]. The increased proportion of nonsynonymous substitutions at this locus (*d*_N_/*d*_S_ > 1) suggests that positive selection drives the evolution of sequence variation at this locus [79]. In several taxa, the observed positive selection has been interpreted as resulting from pathogen-driven selection, leading to extremely high intra-population genetic diversity [11,80,81,82]. This may be associated with the diverse EEHV types reported in Thailand [25,26]. The coexistence of balancing and positive selection is consistent with the hypothesis of selection patterns on the ABR of the second exon of *DQA* in the two elephant genera, *Elephas* and *Loxodonta* [41]. Balancing selection preserves a diverse set of alleles critical for immune function, whereas positive selection drives adaptive changes at specific sites to counter evolving pathogens. This dual mode of selection maintains the extraordinary polymorphism characteristic of MHC loci across vertebrates [78,83,84]. Furthermore, the finding aligns with the role of MHC class II genes, such as *DQA*, in mediating host–pathogen interactions, where diversifying selection maintains allelic variations to cope with diverse infectious agents [85,86].

At the codon level, site-specific selection revealed by MEME, FEL, and FUBAR revealed a combination of episodic diversifying selection and neutral selection at specific codons (11, 14, 18, 19, and 24; *p* < 0.001), reflecting the evolutionary balance between generating diversity to recognize a broad array of pathogens and preserving essential molecular functions [87]. This mixed pattern highlights the complex selective landscape of the *DQA* gene, in which certain residues are adaptively evolving to diversify antigen recognition, whereas others are conserved to maintain the structural or functional integrity of MHC molecules [87]. Such site-specific selection is consistent with previous studies on the selection pattern of MHC genes in elephants and other mammals, which often show heterogeneous selection patterns across codons due to varying functional constraints and pathogen-driven adaptation [41,42]. Alternatively, the observed mixed selection pattern may reflect methodological differences among the detection algorithms. In addition, the fragment analyzed in this study mainly corresponds to exon 2 of the *DQA* gene, which largely overlaps with the peptide-binding region (PBR). Due to this limited sequence coverage, it was not feasible to perform separate neutrality tests between PBR and non-PBR codons. Codon-based analyses (MEME, FEL, and FUBAR), however, consistently detected positively selected sites (codons 11, 14, 18, 19, and 24) within the predicted antigen-binding region. Such a finding supports the expectation that the PBR is subject to stronger diversifying selection than non-PBR sites, which is consistent with previous observations in mammalian MHC genes [41,42,59,60]. FEL assumes constant selective pressure across all sites, whereas MEME is designed to detect episodic selection [51,52]. FUBAR, similar to FEL, assumes constant selection, but is more sensitive to weak positive selection signals [51]. One possible interpretation is that these codons are generally under neutral selection but have experienced episodes of diversifying selection. These findings are, however, based on short fragments from a single locus, and thus should be interpreted cautiously as they might be influenced by the limited number of alleles in each population [57,88]

### 4.3. Implications for Thai Elephant Management

Understanding MHC diversity and selection signatures is critical for enhancing disease resilience in captive populations. Detecting alleles under positive selection can guide functional immunogenetic studies and inform selective breeding strategies that strengthen immune responses while preserving the overall genetic diversity [89,90]. Integrating these alleles into breeding management plans may improve fitness and adaptive capacity, particularly in the face of climate-driven changes in pathogen pressure. Individual transfer between captive populations is a common management practice for captive elephants in Thailand [91]. To optimize such transfers, consideration of MHC allelic diversity is essential to enhance disease resistance within the captive population. For instance, based on maternal and biparental genetic backgrounds [38], individuals from the NEI population possessing maternal lineages β3 and unclassified haplogroups, along with DQA alleles Elma-DQA*TH3, Elma-DQA*TH4, and Elma-DQA*TH8, could be introduced into the MEP and BCEP populations. Such targeted transfers may improve disease resistance and promote overall genetic diversity within captive populations. Data on genetic structure and MHC allele distribution can aid in identifying populations that harbor unique alleles and/or exhibit strong signals of selection, which may be prioritized as “immune variability reservoirs” for conservation purposes [92]. Given these results, the NEI population may serve as a reservoir for immune diversity. This information, supplemented by future data from wild populations, will provide insights into captive and wild elephant management. It will ultimately help prevent genetic homogenization between wild and captive populations and their response to diversifying pathogens and diseases under climate change scenarios [12,92].

## 5. Conclusions

Understanding the genetic diversity and molecular characteristics of MHC *DQA* is essential for elucidating host–pathogen dynamics and ultimately enhancing disease resilience in captive elephant populations. In this study, we investigated genetic variation in a partial exon 2 fragment of the *DQA* gene across three captive elephant camps in Northern Thailand. We established a high allelic diversity, including population-specific alleles, suggesting the influence of localized selective pressures in shaping MHC allele distributions. The observed mixed selection patterns further underscore the complex evolutionary landscape of this locus, reflecting the interplay between pathogen-driven positive selection and functional constraints. Nonetheless, since these findings were derived from relatively short fragments of single loci, future studies should incorporate additional MHC loci and longer sequence fragments as well as include samples from wild populations. This will gain a more comprehensive understanding of immunogenetic variation and pathogen-mediated selection in elephants. Our findings provide novel and valuable insights into the immunogenetic architecture of captive Thai elephants and contribute to a broader understanding of their adaptive immune responses. Such knowledge is critical for the development of conservation strategies and health management practices aimed at improving disease resilience in both captive and wild elephant populations.

## Figures and Tables

**Table 1:** Sequence diversity of partial exon 2 of the *DQA* gene in captive Thai elephants.

Population	N ^1^	*N*_a_ ^2^	*AR* ^3^	*π* ^4^
NEI ^a^	39	8	0.805 ± 0.015	0.032 ± 0.00083
BCEP ^b^	41	5	0.746 ± 0.013	0.027 ± 0.00092
MEP ^c^	40	6	0.799 ± 0.015	0.029 ± 0.00144
Overall	123	8	0.786 ± 0.007	0.030 ± 0.00064

^1^ Number of individuals (N), ^2^ Number of alleles (*N*_a_), ^3^ allelic richness (*AR*), ^4^ nucleotide diversity (*π*), ^a^ National Elephant Institute of Thailand (NEI), ^b^ Baan Chang Elephant Park (BCEP), ^c^ Maetaeng Elephant Park (MEP).

**Table 2:** Neutrality test and rates of synonymous (*d*_S_) and nonsynonymous (*d*_N_) substitutions in the nucleotide sequences of the *DQA* gene in captive Thai elephants.

Population	Tajima’s *D*	Fu and Li *D*	Fu and Li *F*	Nei–Gojobori’s Method			Li–Wu–Luo’s Method		
				*d*_N_	*d*_S_	*d*_N_/*d*_S_ (ω)	*d*_N_	*d*_S_	*d*_N_/*d*_S_ (ω)
NEI ^a^	1.432 ^ns^	0.936 ^ns^	1.321 ^ns^	0.033	0.006	5.5	0.015	0.032	2.1
BCEP ^b^	1.631 ^ns^	1.404 ^ns^	1.773 *	0.032	0.008	3.9	0.008	0.032	4.0
MEP ^c^	1.299 ^ns^	0.872 ^ns^	1.212 ^ns^	0.033	0.008	4.1	0.015	0.034	2.3
Overall	1.794 ^ns^	1.454 ^ns^	1.909 *	0.030	0.008	3.8	0.013	0.033	2.8

*, *p* < 0.05; ^ns^, not significant; ^a^ National Elephant Institute of Thailand (NEI); ^b^ Baan Chang Elephant Park (BCEP); ^c^ Maetaeng Elephant Park (MEP).

## Data Availability

The allele sequences found in this study were deposited in the National Center for Biotechnology Information (NCBI) (https://www.ncbi.nlm.nih.gov/, accessed on 9 September 2025) (accession numbers: SRX30398467–SRX30398474).

## Supplementary Materials

The following supporting information can be downloaded at: https://www.mdpi.com/article/10.3390/genes16101180/s1, Figure S1: Bayesian phylogenetic tree of amino acids in partial exon 2 *DQA* gene alleles for captive Thai elephant. The values above the branches represent posterior probability. Scale shows substitutions per site; Table S1: Elephant individual used in this study; Table S2: Variable sites of partial exon 2 *DQA* gene alleles found in this study; Table S3: Mutation types and their locations in the partial fragments of *DQA* gene exon 2. The fragments of captive elephant compared with the reference sequence (accession number: CM065237); Table S4: Allelic frequency of the partial exon 2 *DQA* gene alleles found in this study; Table S5: Detailed site-by-site result from Mixed Effects Model of Evolution (MEME) analysis; Table S6: Detailed site-by-site result from Fixed Effects Likelihood (FEL) analysis; Table S7: Detailed site-by-site result from Fast Unconstrained Bayesian AppRoximation (FUBAR) analysis.

## Abbreviations

The following abbreviations are used in this manuscript:

MHC	Major histocompatibility complex
IUCN	International Union for Conservation of Nature and Natural Resources
EEHV	Elephant endotheliotropic herpesvirus
MEP	Maetaeng Elephant Park
BCEP	Baan Chang Elephant Park
NEI	National Elephant Institute of Thailand
DOC	Degree of change
FUBAR	Fast Unconstrained Bayesian AppRoximation
FEL	Fixed effect likelihood
MEME	Mixed Effects Model of Evolution
ABR	Antigen-binding-region
