# Supplementary material for: Evidence of Mixed Selection Acting on the MHC Class II DQA Gene in Captive Thai Elephant Populations
Source: Genes (Basel). 2025 Oct 10;16(10):1180. doi: 10.3390/genes16101180 (PMC12563602; doi:10.3390/genes16101180)
Supplement: Supplementary file 1 [file genes-16-01180-s001.zip › genes-3910231-supplementary.pdf]

Supplementary Files

Supplementary Figures

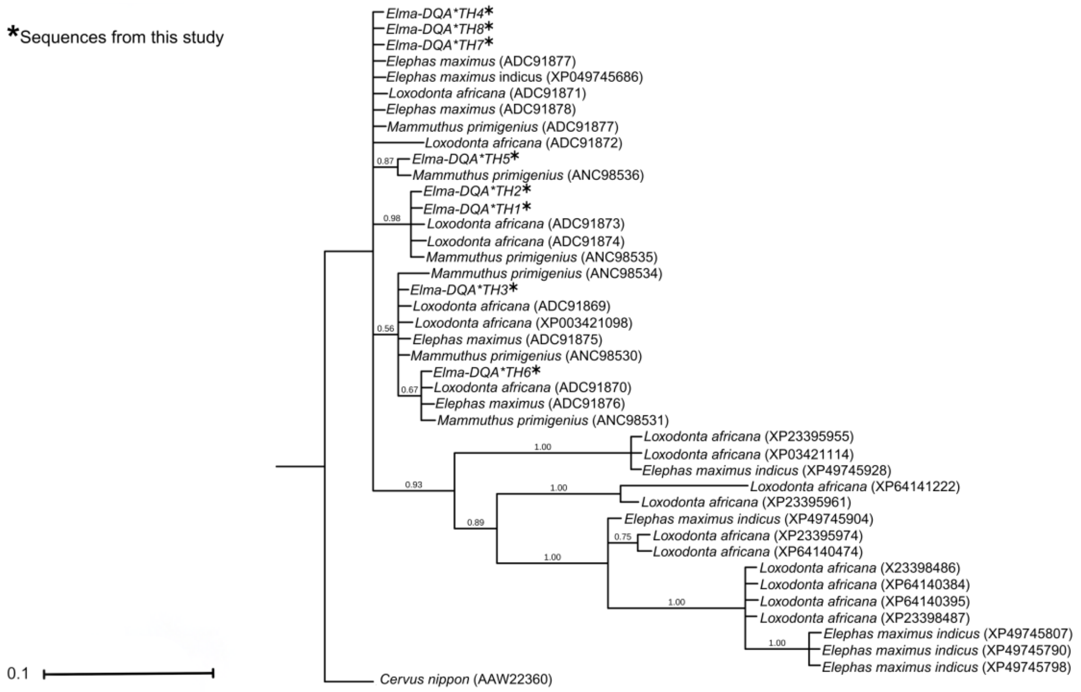

Figure S1 Bayesian phylogenetic tree of amino acids in partial exon 2 *DQA* gene alleles for captive Thai elephant. The values above the branches represent posterior probability. Scale shows substitutions per site.

Supplementary Tables

Table S1 Elephant individual used in this study

No.	Abbreviation/ Code	Sex	Locality
1	EMAf05	female	NEI, Lampang
2	EMAf09	female	NEI, Lampang
3	EMAf10	female	NEI, Lampang
4	EMAf11	female	NEI, Lampang
5	EMAf13	female	NEI, Lampang
6	EMAf17	female	NEI, Lampang
7	EMAf29	female	NEI, Lampang
8	EMAf30	female	NEI, Lampang
9	EMAf31	female	NEI, Lampang
10	EMAf32	female	NEI, Lampang
11	EMAf33	female	NEI, Lampang
12	EMAf34	female	NEI, Lampang
13	EMAf35	female	NEI, Lampang
14	EMAf36	female	NEI, Lampang
15	EMAf37	female	NEI, Lampang
16	EMAf38	female	NEI, Lampang
17	EMAf39	female	NEI, Lampang
18	EMAm01	male	NEI, Lampang
19	EMAm02	male	NEI, Lampang
20	EMAm03	male	NEI, Lampang
21	EMAm04	male	NEI, Lampang
22	EMAm05	male	NEI, Lampang
23	EMAm07	male	NEI, Lampang
24	EMAm08	male	NEI, Lampang
25	EMAm09	male	NEI, Lampang
26	EMAm10	male	NEI, Lampang
27	EMAm21	male	NEI, Lampang
28	EMAm22	male	NEI, Lampang
29	EMAm23	male	NEI, Lampang
30	EMAm24	male	NEI, Lampang
31	EMAm25	male	NEI, Lampang
32	EMAm26	male	NEI, Lampang
33	EMAm30	male	NEI, Lampang
34	EMAlm13	male	NEI, Lampang
35	EMAm14	male	NEI, Lampang
36	EMAm16	male	NEI, Lampang
37	EMAm17	male	NEI, Lampang
38	EMAm18	male	NEI, Lampang
39	EMAm20	male	NEI, Lampang
40	EMAIm01	male	BCEP, Chiang Mai

No.	Abbreviation/ Code	Sex	Locality
41	EMAIm02	male	BCEP, Chiang Mai
42	EMAIm04	male	BCEP, Chiang Mai
43	EMAIm6	male	BCEP, Chiang Mai
44	EMAIm7	male	BCEP, Chiang Mai
45	EMAIm8	male	BCEP, Chiang Mai
46	EMAIm12	male	BCEP, Chiang Mai
47	EMAIf17	female	BCEP, Chiang Mai
48	EMAIf18	female	BCEP, Chiang Mai
49	EMAIf19	female	BCEP, Chiang Mai
50	EMAIf20	female	BCEP, Chiang Mai
51	EMAIf22	female	BCEP, Chiang Mai
52	EMAIf23	female	BCEP, Chiang Mai
53	EMAIf24	female	BCEP, Chiang Mai
54	EMAIf26	female	BCEP, Chiang Mai
55	EMAIf27	female	BCEP, Chiang Mai
56	EMAIf28	female	BCEP, Chiang Mai
57	EMAIf29	female	BCEP, Chiang Mai
58	EMAIf30	female	BCEP, Chiang Mai
59	EMAIf31	female	BCEP, Chiang Mai
60	EMAIf32	female	BCEP, Chiang Mai
61	EMAIf33	female	BCEP, Chiang Mai
62	EMAIf35	female	BCEP, Chiang Mai
63	EMAIf36	female	BCEP, Chiang Mai
64	EMAIf37	female	BCEP, Chiang Mai
65	EMAIf38	female	BCEP, Chiang Mai
66	EMAIf39	female	BCEP, Chiang Mai
67	EMAIf40	female	BCEP, Chiang Mai
68	EMAIf41	female	BCEP, Chiang Mai
69	EMAIf42	female	BCEP, Chiang Mai
70	EMAIf43	female	BCEP, Chiang Mai
71	EMAIf45	female	BCEP, Chiang Mai
72	EMAIf46	female	BCEP, Chiang Mai
73	EMAIf47	female	BCEP, Chiang Mai
74	EMAIf48	female	BCEP, Chiang Mai
75	EMAIf49	female	BCEP, Chiang Mai
76	EMAIf50	female	BCEP, Chiang Mai
77	EMAIf51	female	BCEP, Chiang Mai
78	EMAIf52	female	BCEP, Chiang Mai
79	EMAIf53	female	BCEP, Chiang Mai
80	EMAIf54	female	BCEP, Chiang Mai
81	EMAFf03	female	MEP, Chiang Mai
82	EMAFf04	female	MEP, Chiang Mai

No.	Abbreviation/ Code	Sex	Locality
83	EMAFf05	female	MEP, Chiang Mai
84	EMAFf06	female	MEP, Chiang Mai
85	EMAFf07	female	MEP, Chiang Mai
86	EMAFf08	female	MEP, Chiang Mai
87	EMAFf09	female	MEP, Chiang Mai
88	EMAFf10	female	MEP, Chiang Mai
89	EMAFf11	female	MEP, Chiang Mai
90	EMAFf12	female	MEP, Chiang Mai
91	EMAFf13	female	MEP, Chiang Mai
92	EMAFf14	female	MEP, Chiang Mai
93	EMAFf15	female	MEP, Chiang Mai
94	EMAFf16	female	MEP, Chiang Mai
95	EMAFf17	female	MEP, Chiang Mai
96	EMAFf18	female	MEP, Chiang Mai
97	EMAFf20	female	MEP, Chiang Mai
98	EMAFf21	female	MEP, Chiang Mai
99	EMAFf22	female	MEP, Chiang Mai
100	EMAFf23	female	MEP, Chiang Mai
101	EMAFf24	female	MEP, Chiang Mai
102	EMAFf25	female	MEP, Chiang Mai
103	EMAFf26	female	MEP, Chiang Mai
104	EMAFf27	female	MEP, Chiang Mai
105	EMAFf28	female	MEP, Chiang Mai
106	EMAFf29	female	MEP, Chiang Mai
107	EMAFf30	female	MEP, Chiang Mai
108	EMAFf31	female	MEP, Chiang Mai
109	EMAFf32	female	MEP, Chiang Mai
110	EMAFf34	female	MEP, Chiang Mai
111	EMAFf35	female	MEP, Chiang Mai
112	EMAFm01	male	MEP, Chiang Mai
113	EMAFm02	male	MEP, Chiang Mai
114	EMAFm04	male	MEP, Chiang Mai
115	EMAFm05	male	MEP, Chiang Mai
116	EMAFm06	male	MEP, Chiang Mai
117	EMAFm07	male	MEP, Chiang Mai
118	EMAFm08	male	MEP, Chiang Mai
119	EMAFm09	male	MEP, Chiang Mai
120	EMAFm10	male	MEP, Chiang Mai

Table S2 Variable sites of partial exon 2 *DQA* gene alleles found in this study

Position	95799912	95799913	95799945	95799954	95799964	95799965	95799967	95799968	95799969	95799982	95799983
Elephas maximus (CM065237)	G	G	G	T	G	C	A	G	T	G	G
*Elma-DQA*TH1*	.	A	.	.	.	.	G	.	A	.	.
*Elma-DQA*TH2*	T	A	.	.	.	.	G	.	A	.	.
*Elma-DQA*TH3*	.	.	A	.	.	A	.	.	.	.	.
*Elma-DQA*TH4*	.	.	.	.	.	.	.	.	.	.	.
*Elma-DQA*TH5*	.	.	.	.	A	.	.	.	.	.	.
*Elma-DQA*TH6*	.	.	.	.	.	A	.	.	.	C	T
*Elma-DQA*TH7*	.	.	.	C	.	.	T	C	G	.	.
*Elma-DQA*TH8*	T	.	.	C	.	.	T	C	G	.	.

Table S3 Mutation types and their locations in the partial fragments of *DQA* gene exon 2. The fragments of captive elephant compared with the reference sequence (accession number: CM065237)

No	Allele	Position of mutation and nucleotide substitution	Mutation type	Amino acid change (nucleotide substitution)
1	*Elma-DQA*TH1* and *Elma-DQA*TH2*	95,799,913G>A	Missense	Asp>Asn (GAC to AAC)
2	*Elma-DQA*TH3*	95,799,945G>A	Silent	-
3	*Elma-DQA*TH7* and *Elma-DQA*TH8*	95,799,954T>C	Silent	-
4	*Elma-DQA*TH5*	95,799,964G>A	Missense	Ala>Thr (GCA to ACA)
5	*Elma-DQA*TH3* and *Elma-DQA*TH6*	95,799,965C>A	Missense	Ala>Glu (GCA to GAA)
6	*Elma-DQA*TH7* and *Elma-DQA*TH8*	95,799,967A>T	Silent	
7	*Elma-DQA*TH1* and *Elma-DQA*TH2*	95,799,967A>G	Missense	Ser>Gly (AGT to GGA)
8	*Elma-DQA*TH7* and *Elma-DQA*TH8*	95,799,968G>C	Silent	-
9	*Elma-DQA*TH7* and *Elma-DQA*TH8*	95,799,969T>G	Silent	-
10	*Elma-DQA*TH1* and *Elma-DQA*TH2*	95,799,969T>A	Missense	Ser>Gly (AGT to GGA)
11	*Elma-DQA*TH6*	95,799,982G>C	Missense	Gly>Leu (GGT to CTT)
12	*Elma-DQA*TH6*	95,799,983G>T	Missense	Gly>Leu (GGT to CTT)

Table S4 Allelic frequency of the partial exon 2 DQA gene alleles found in this study

Population	N	*Elma-DQA*TH1*	*Elma-DQA*TH2*	*Elma-DQA*TH3*	*Elma-DQA*TH4*	*Elma-DQA*TH5*	*Elma-DQA*TH6*	*Elma-DQA*TH7*	*Elma-DQA*TH8*
NEI[a]	39	0.233	0.267	0.012	0.012	0.174	0.186	0.093	0.023
BCEP[b]	41	0.289	0.049	0.000	0.000	0.289	0.289	0.085	0.000
MEP[c]	40	0.177	0.165	0.000	0.000	0.266	0.266	0.127	0.000
Overall	123	0.244	0.140	0.003	0.003	0.251	0.254	0.098	0.007

[a]National Elephant Institute of Thailand (NEI), [b]Baan Chang Elephant Park (BCEP), [c]Maetaeng Elephant Park (MEP).

Table S5 Detailed site-by-site result from Mixed Effects Model of Evolution (MEME) analysis

Codon	α	β1	p1	β2	p2	β3	p3	β+	q	Class	Selection type
1	1	0	0	1	0	0	0	0	0	0	Invariable
1	2	0	0	1	0	0	0	0	0	0	Invariable
1	3	0	0	1	0	0	0	0	0	0	Invariable
1	4	0	0	1	0	0	0	0	0	0	Invariable
1	5	0	0	1	0	0	0	0	0	0	Invariable
1	6	0	0	1	0	0	0	0	0	0	Invariable
1	7	0	0	1	0	0	0	0	0	0	Invariable
1	8	0	0	1	0	0	0	0	0	0	Invariable
1	9	0	0	1	0	0	0	0	0	0	Invariable
1	10	0	0	1	0	0	0	0	0	0	Invariable
1	11	20.108	0	0	0	0	6.357	0	1.457	0	Diversifying
1	12	0	0	1	0	0	0	0	0	0	Invariable
1	13	0	0	1	0	0	0	0	0	0	Invariable
1	14	203.839	0	0	0	0	51.004	0	6.758	0	Diversifying
1	15	0	0	1	0	0	0	0	0	0	Invariable
1	16	0	0	1	0	0	0	0	0	0	Invariable
1	17	0	0	1	0	0	0	0	0	0	Invariable
1	18	0.044	0.014	0	0.024	0	0.027	0	141.678	0	Diversifying
1	19	0.224	0	0	0.224	0	0	0	13585.22	0	Diversifying
1	20	0	0	1	0	0	0	0	0	0	Invariable
1	21	0	0	1	0	0	0	0	0	0	Invariable
1	22	0	0	1	0	0	0	0	0	0	Invariable
1	23	0	0	1	0	0	0	0	0	0	Invariable
1	24	0	0	0	0	0	0	0	194.173	0	Diversifying
1	25	0	0	1	0	0	0	0	0	0	Invariable

Codon	α	β1	p1	β2	p2	β3	p3	β+	q	Class	Selection type
1	26	0	0	1	0	0	0	0	0	0	Invariable
1	27	0	0	1	0	0	0	0	0	0	Invariable
1	28	0	0	1	0	0	0	0	0	0	Invariable
1	29	0	0	1	0	0	0	0	0	0	Invariable
1	30	0	0	1	0	0	0	0	0	0	Invariable
1	31	0	0	1	0	0	0	0	0	0	Invariable
1	32	0	0	1	0	0	0	0	0	0	Invariable
1	33	0	0	1	0	0	0	0	0	0	Invariable
1	34	0	0	1	0	0	0	0	0	0	Invariable
1	35	0	0	1	0	0	0	0	0	0	Invariable
1	36	0	0	1	0	0	0	0	0	0	Invariable
1	37	0	0	1	0	0	0	0	0	0	Invariable
1	38	0	0	1	0	0	0	0	0	0	Invariable
1	39	0	0	1	0	0	0	0	0	0	Invariable
1	40	0	0	1	0	0	0	0	0	0	Invariable

α, synonymous substitution rate; β, nonsynonymous substitution rate; p, proportion of branches evolving under selection; q, empirical bayes factor

Table S6 Detailed site-by-site result from Fixed Effects Likelihood (FEL) analysis

Codon	α	β	α = β	LRT	*p*-value	Total branch length	class
1	0	0	0	0	1	0	Invariable
2	0	0	0	0	1	0	Invariable
3	0	0	0	0	1	0	Invariable
4	0	0	0	0	1	0	Invariable
5	0	0	0	0	1	0	Invariable
6	0	0	0	0	1	0	Invariable
7	0	0	0	0	1	0	Invariable
8	0	0	0	0	1	0	Invariable
9	0	0	0	0	1	0	Invariable
10	0	0	0	0	1	0	Invariable
11	7.345	0	3.351	1.466	0.2261	0.382	Neutral
12	0	0	0	0	1	0	Invariable
13	0	0	0	0	1	0	Invariable
14	76.242	0	3.573	5.876	0.0153	0.407	Neutral
15	0	0	0	0	1	0	Invariable
16	0	0	0	0	1	0	Invariable
17	0	0	0	0	1	0	Invariable
18	0	9.609	6.078	1.7	0.1923	0.693	Neutral
19	72.898	11.17	16.227	1.697	0.1927	1.85	Neutral
20	0	0	0	0	1	0	Invariable
21	0	0	0	0	1	0	Invariable
22	0	0	0	0	1	0	Invariable
23	0	0	0	0	1	0	Invariable
24	0	6.315	4.552	1.261	0.2615	0.519	Neutral
25	0	0	0	0	1	0	Invariable
26	0	0	0	0	1	0	Invariable
27	0	0	0	0	1	0	Invariable
28	0	0	0	0	1	0	Invariable
29	0	0	0	0	1	0	Invariable
30	0	0	0	0	1	0	Invariable
31	0	0	0	0	1	0	Invariable
32	0	0	0	0	1	0	Invariable

Codon	α	β	α = β	LRT	*p*-value	Total branch length	class
33	0	0	0	0	1	0	Invariable
34	0	0	0	0	1	0	Invariable
35	0	0	0	0	1	0	Invariable
36	0	0	0	0	1	0	Invariable
37	0	0	0	0	1	0	Invariable
38	0	0	0	0	1	0	Invariable
39	0	0	0	0	1	0	Invariable
40	0	0	0	0	1	0	Invariable

α, synonymous substitution rate; β, nonsynonymous substitution rate; LRT, likelihood ratio test

Table S7 Detailed site-by-site result from Fast Unconstrained Bayesian AppRoximation (FUBAR) analysis

Codon	α	β	β-α	Prob[α>β]	Prob[α<β]	Bayes Factor[α<β]
1	3.17	2.21	-0.96	0.332	0.615	1.816
2	0.973	0.864	-0.109	0.477	0.464	0.983
3	1.943	0.693	-1.25	0.528	0.415	0.805
4	1.944	0.725	-1.219	0.524	0.418	0.818
5	1.944	0.725	-1.219	0.524	0.418	0.818
6	1.943	0.693	-1.25	0.528	0.415	0.805
7	1.117	0.776	-0.341	0.495	0.446	0.915
8	1.137	0.704	-0.432	0.5	0.44	0.895
9	5.115	0.706	-4.409	0.586	0.363	0.646
10	0.956	0.738	-0.218	0.489	0.451	0.934
11	8.938	0.881	-8.057	0.82	0.144	0.192
12	1.117	0.802	-0.315	0.492	0.449	0.926
13	5.109	0.621	-4.488	0.598	0.351	0.615
14	27.028	0.708	-26.319	0.95	0.04	0.047
15	3.109	0.594	-2.515	0.571	0.374	0.679
16	1.398	0.777	-0.62	0.504	0.438	0.884
17	3.561	0.644	-2.917	0.57	0.376	0.685
18	1.218	12.615	11.398	0.048	0.926	14.233
19	23.309	18.32	-4.989	0.485	0.39	0.726
20	3.561	0.644	-2.917	0.57	0.376	0.685
21	3.11	0.627	-2.483	0.566	0.38	0.696
22	1.092	0.803	-0.289	0.489	0.452	0.936
23	1.398	0.818	-0.58	0.5	0.441	0.898
24	1.164	7.523	6.359	0.078	0.887	8.933
25	1.091	0.657	-0.434	0.506	0.434	0.871
26	0.973	0.864	-0.109	0.477	0.464	0.983
27	1.509	0.624	-0.885	0.527	0.414	0.804
28	3.112	0.664	-2.448	0.56	0.385	0.713
29	2.781	0.647	-2.134	0.556	0.388	0.722
30	1.117	0.708	-0.409	0.502	0.438	0.887
31	1.174	0.669	-0.505	0.508	0.432	0.865
32	1.138	0.852	-0.286	0.485	0.456	0.952
33	1.397	0.736	-0.661	0.508	0.433	0.869
34	1.943	0.693	-1.25	0.528	0.415	0.805
35	3.112	0.679	-2.434	0.558	0.388	0.719
36	1.137	0.776	-0.361	0.492	0.449	0.925
37	1.117	0.774	-0.343	0.494	0.446	0.916
38	1.397	0.736	-0.661	0.508	0.433	0.869
39	1.124	0.799	-0.325	0.493	0.447	0.921
40	1.944	0.755	-1.189	0.521	0.422	0.83

α, synonymous substitution rate; β, nonsynonymous substitution rate
